# Improving learning by playing with an app: The FantaTraining ^® ^experience with anesthesia trainees.

**DOI:** 10.12688/mep.19148.1

**Published:** 2022-05-26

**Authors:** Giovanni Gibiino, Emanuele Capogna, Matteo Velardo, Angelica Del Vecchio, Pier Luigi Ingrassia, Giorgio Capogna

**Affiliations:** 1 European e-Learning School in Obstetric Anesthesia (EESOA), Rome, Italy; 2 Simulation Center (CeSi), Professional Socio-Health Center of Lugano (CPS), Lugano, Switzerland

**Keywords:** gamification, app, anesthesia, education

## Abstract

**Background:** FantaTraining ^®^ is an app that simulates a football league. Each participant represents a team, and the game is played with the opposing team by answering a questionnaire. In the intervals between games, participants can practice by consulting the educational material (films, short texts, or slides) in the app. Various prizes are offered to the winners of the championship. In this study, we aimed to evaluate whether the use of the FantaTraining ^®^ app could improve the learning of anesthesia trainees registered in an online obstetric anesthesia course.

**Methods:** The study involved 282 trainees in anesthesia, from five Italian universities, registered in the Online Obstetric Anesthesia Course (OOAC) who were given the app. They were randomly allocated into two groups according to whether the app had been enabled to allow the participant to play the league (study group, n=137), or not (control group, n=145). All the trainees underwent entry and final tests, consisting of the same 40 multiple choice questions, respectively before and after completing the OOAC course.

**Results:** There were no differences in the scores obtained in the pre-course test between the groups. The mean score obtained in the final test was significantly greater than that obtained in the entry one in both groups (P<0.05) but the final test score obtained by the participants of the study group was significantly greater than that obtained by the control group. (P<0.001), regardless of the university of origin and year of specialization. Trainees stated that the app had helped their study, improving understanding and motivation, without increasing the intensity of study.

**Conclusions:** Using the FantaTraining ^®^ app greatly improved trainees’ final exam performance after the online obstetric anesthesia course. The FantaTraining ^®^ app seems a promising tool to improve learning outcomes by strengthening learning behaviors and attitudes towards learning.

## Introduction

In the recent years, games have been reported to be able to increase learning outcomes in many fields of medical education, thus leading to the introduction of the term “gamification” ( Koivisto & Hamari, 2019; Seaborn & Fels, 2015). This term refers to the inclusion of game elements, such as points and rewards, to tasks as incentives for participants in order to facilitate learning ( Caponetto *et al.,* 2014; Koivisto & Hamari, 2019; Majuri *et al.,* 2018; Osatuyi *et al.,* 2018).

There are many games and gamification options. Quizzes, for example, being one of the most common teaching gamification, allowing participants to verify their knowledge by using different platforms, such as on the web or apps. Different strategies have been employed: the challenge based ( Koivisto & Hamari, 2019), the immersion based, which involves the user in a story ( Concannon *et al.,* 2019) and the social-based gamification, which is based on competition and collaboration ( Romero, 2017).

Gamified activities aim to improve the students’ internal and external motivation based on the role of rewards and incentives ( Richter *et al.,* 2015). In addition, gamification has also been associated with the goal-setting theory. This theory explains that there are four factors which can affect the students’ performance: their commitment towards the goal, the feedback they receive, the complexity of the activity, and the situational limits ( Landers, 2014; Locke & Latham, 2002; Locke & Latham, 2006). Basically, gamification requires a challenge, some progress feedback, the knowledge of the levels of achievement, and some type of competition ( Huang & Hew, 2018). Another theory linked to gamification is the flow theory, where the good psychological and physical state of the participant may optimize enjoyment and engagement. Consistent with this theory, gamification would require specific and easily comprehensible goals, instantaneous feedback, accomplishment indicators, and a satisfactory balance between challenges, the participant’s skills, and the seeming value of the activity ( Huang & Hew, 2018).

Game accessible via mobile phones from the app store has been used to deliver simulation courses to medical students ( Smith & Holland, 2021).

Brain Refresher Lab ( https://www.brainrefreshlab.com) has recently developed an app called FantaTraining ^®^, (available on Apple store and Google Play) which is based on the gamification theories and that has been successfully used in marketing and business training. This app simulates a football league in which participants sign up and play matches with other registered participants to win the league. There is training between matches and prizes for the winners. Each participant represents a team, and the game is played with the opposing team by answering a questionnaire that checks the number of correct answers on a chosen topic. In the intervals between games, participants can practice by consulting the educational material (films, short texts, or slides) in the app. Various prizes are offered to the winners of the championship.

In this study we aimed to evaluate whether the use of the FantaTraining ^®^ app could improve the learning of anesthesia trainees registered in an online obstetric anesthesia course.

## Methods

### Ethical statement

This study was carried out according to the Declaration of Helsinki. In our region, simulation centers do not have access to a formal ethical approval process, and it was not possible to submit the study under a different ethics committee in Italy. However, the study followed the Healthcare Simulationist Code of Ethics supported by the Society for Simulation in Healthcare ( Healthcare Simulationist Code of Ethics, 2018) The study was low-risk and non-clinical, with no direct contact with participants as all data collection was doing online. The trainees participating were volunteers, researchers ensured that those taking part in the research would not be caused distress, all the participants' personal and other data were completely anonymized, and all the investigators had no conflict of interest and were not involved in any of the participants' university teaching programs. Written informed consent was obtained from all participants prior to participation in the study.

### Study design

The study took place online from October to December 2021.

It involved 300 trainees in anesthesia, from five Italian universities, registered on the Online Obstetric Anesthesia Course (OOAC) of the European School of Obstetric Anesthesia and Simulation Center (EESOA, Rome) who were offered voluntary enrollment in this study. This three-month, annual course consists of seven online lessons of 90 minutes each on subjects of obstetric anesthesia.

After registering in the EESOA OOAC and before the start of the lessons, all the anesthesia trainees who had given their consent to participate in the study were sent the FantaTraining ^®^ app. For the purpose of the study the FantaTraining ^®^ app was assembled in such a way that it contained, for each of the seven online lessons, both the additional learning materials (short video clips, tests, and slides) and the multiple choice quizzes representing the football matches to be played.

The app requires the participant to play a game (answer quizzes on the topics covered in the lesson in competition with another participant) in a set time interval between lessons for a predefined duration and leaves the participant free to consult the teaching material ("coaching") until shortly before the game. The app automatically calculates a provisional ranking for each game session, which is made known to the players, who thus know the score of the opponents they are challenging.

Among the trainees enrolled in the study 18 declined, and therefore 282 were randomized (simple randomization) and allocated into two groups according to whether the app had been enabled to allow the participant to play the league (study group, n=137), or not (control group, n=145).

In practice, the participants belonging to the study group were able to use the app both to consult further teaching materials and to play the championship, while those in the control group could only consult the teaching materials.

The trainees in the control group were told that the top five in the league would be offered a free simulation course. To encourage participation and play ( Richter *et al.,* 2015), all those who completed the course were offered a book on obstetric anesthesia.

All the trainees underwent to entry and final tests, consisting of the same 40 multiple choice questions, respectively before and after completing the OOAC course. Every correct answer was given a score of one, therefore the maximum score obtainable was 40.

In addition, the study group participants completed a satisfaction questionnaire ( Table 1) after having finished the course and given the final test.

**Table 1.** Satisfaction questionnaire.

Likert Scale (1-5): 1 Strongly disagree/ 2 Disagree / 3 Neutral/ 4 Agree/ 5 Strongly agree • Would you have studied with the same intensity without the App? (intensity) • Has the App been helpful in motivating you to study? (motivation) • Has the App been helpful to you as a study support? (study support) • Was the App helpful in understanding the study material? (understanding)

### Statistics

Statistical software R version 4.0.2 was used for data processing and the generation of tables and figures. For quantitative variables (test score and time) appropriate descriptive statistics (i.e., mean, standard deviation, median, quartiles) were calculated and results reported as mean ± SD or median ±MAD. Absolute and percentage frequency distributions were presented for qualitative variables (age, gender, university, and year of specialization). Kolmogorov-Smirnov and Shapiro-Wilk normality tests were applied to test the normality hypothesis.

Parametric statistical models such as repeated measure mixed effect analysis of variance (ANOVA) (post-hoc Tukey), and linear regression, were applied to dependent variables, and assumptions about residuals were tested for homoskedasticity, independence, and identical distribution.

For questionnaires that included Likert scale response modes, agreement indicators were calculated to assess the degree of agreement of the participants. There was maximum agreement towards the worst judgment when the indicator is equal to 0, while there was maximum agreement towards the best judgment when it is equal to 1 (disagreement among respondents returns an indicator equal to 0.5).

Power analysis was carried out on the primary endpoint of the study (mixed effect repeated measure ANOVA) which provided for a sample size of 220 to ensure a level of significance of 95% and a test power of 80%.

In addition, for the analysis of the questionnaire, setting a confidence level of 90% and a margin of error of 5%, a sample size of 268 residents was required.

## Results

**Figure 1.**
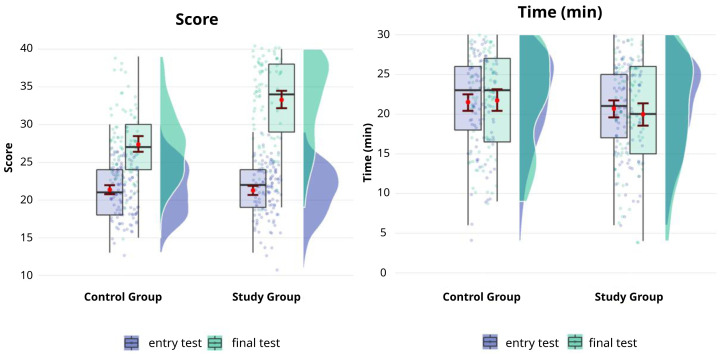
Mean scores before and after the course with or without using of the FantaTraining ^®^ app. Boxplot and distribution of the mean score before and after the course with (study group) or without (control group) playing the championship. The horizontal line displays 25 ^th^, 50 ^th^, and 75 ^th^ percentile, the red point and vertical bar indicate the mean and confidence interval.

The mean age of the participants was 29 (±3) years (N=282). There were no differences in the scores obtained in the pre-course test between the groups ( Figure 1). The mean score obtained in the final test (after the end of the course) was significantly greater than that obtained in the first one (performed before starting the course) in both groups (P<0.05) but the final test score obtained by the participants of the study group was significantly greater than that obtained by the control group. (P<0.001) ( Figure 1), regardless of the university of origin and the year of specialization ( Velardo, 2022). 

There were no differences between the groups in the time elapsed to perform the test ( Figure 1).

Concerning the analysis of the participants who had played the games, the median number of games played was eight out of a possible total of 13.

**Figure 2.**
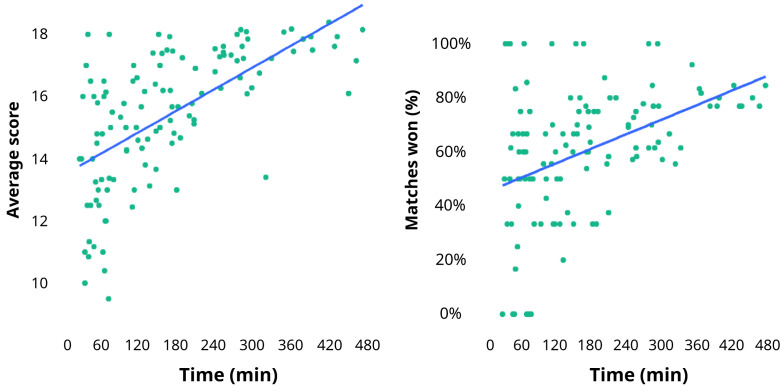
Linear regression model of the correlation between the average score, matches won, and the time of use of the FantaTraining ^®^ app in the study group.

By applying a linear regression model, we observed that the mean score of the games played, and the percentage of matches won were positively correlated with the time of use of the app: the model estimated that for every hour of application use, the percentage of games won can increase by 15% (P<0.05) and the average score can increase by 3.6 (P<0.05) ( Figure 2).

Results of the questionnaire are reported in the Table 2. Trainees stated that the app helped their study, improving understanding and motivation, without increasing the intensity of study.

**Table 2.** Frequency distribution and agreement indicator of questionnaire answers (N=282).

ITEM	Strongly disagree	Disagree	Neutral	Agree	Strongly agree	Agreement indicator
Understanding	1%	2%	13%	43%	41%	0,87
Intensity	14%	32%	35%	14%	4%	0,46
Motivation	1%	7%	17%	37%	39%	0,83
Support to study	1%	2%	13%	34%	50%	0,88

## Discussion

The current young doctors’ generation utilizes games and simulations to create collaborative learning environments. The so called mobile-learning has the potential for social interactivity in gaming scenarios and increased practice problem-solving on different engaging and visually stimulating platforms. Rather than the traditional education and information delivery methods, users can have individualized experiences, track their progress, and study alone or with colleagues adhering to their specific time and location limitations ( Becker, 2019).

In this study the primary source of knowledge was a ‘traditional’ online course, and we employed the app as a teaching support to be used between the online lessons.

Using the FantaTraining ^®^ app greatly improved the trainees’ final exam performance after the online obstetric anesthesia course. Our results are in line with the theories that gamification improves trainees’ engagement and motivation by adding game elements such as instant feedback, earning rewards and tracking challenge completion. Gamification also adjusts the way the brain processes new knowledge through releasing bite-sized portions of information, audio-visual stimulation, short time-lapses and repetitive patterns ( Sailer & Homner, 2020; Shurui Bai *et al.,* 2020; van Gaalen *et al.,* 2021).

The positive results obtained with the FantaTraining app may be explained by the theory of gamified learning ( Landers, 2014) which suggests that instructional content impacts learning outcomes as well as learners’ behavior *.* According to this theory, the goal of gamification is to alter a contextual learner’s behavior or attitude (e.g., engagement), to improve pre-existing instruction.

In addition to an increased learning performance, we also noted high levels of satisfaction reported by our users: trainees affirmed that the app significantly helped their study improving both understanding and motivation, without increasing the intensity of study and this may certainly be considered an additional advantage of the use of this app. This participant opinion was also confirmed by the results obtained by our regression model, which was able to predict a significant improvement in learning performance by increasing the amount of time of use of the app.

## Limitations

Our study has some limitations. It is a preliminary study and, although the sample size is more than sufficient to justify the results, the use of the FantaTraining ^®^ app should be extended to a larger sample in order to better evaluate its effects over time. We hope that it will be included in the educational programs of the universities that have joined the study, so as to be able to better highlight any criticalities of the app, which will probably be highlighted only with its routine use.

## Conclusion

In conclusion, the FantaTraining ^®^ app seems a promising tool to improve learning outcomes by strengthening learning behaviors and attitudes towards learning, and we hope the results of our study may contribute to a better comprehension of the role of new learning methods in health profession education. Further studies are also needed with students from other medical specialties to confirm our positive findings. In particular, it would be interesting to investigate the exclusive use of FantaTraining ^®^ as the primary substitute resource for online classes.

## Data availability

### Underlying data

Harvard Dataverse: Improving learning by playing with an app: The FantaTraining® experience with anesthesia. https://doi.org/10.7910/DVN/TXDS2E. ( Velardo, 2022).

This project contains the following underlying data:

- DB Fantatraining 1. (Anonymized results for control and study group participants on Fanta-Training database.)
- ANOVA by university. (ANOVA results considering university)
- ANOVA by years of specialization. (ANOVA results considering year of specialization)

Data are available under the terms of the Creative Commons Zero “No rights reserved” data waiver (CC0 1.0 Public domain dedication).
