# Supplementary material for: Improving learning by playing with an app: The FantaTraining ® experience with anesthesia trainees
Source: MedEdPublish (2016). 2022 May 26;12:38. [Version 1] doi: 10.12688/mep.19148.1 (PMC9370084; doi:10.12688/mep.19148.1)
Supplement: Peer review history

# Peer review: Reviewer response for version 1

**DOI:** 10.21956/mep.20506.r32055

**Published:** 2022-06-10

**Authors:** Marc Lazarovic

**Affiliations:** 1 Institute for Emergency Medicine, Ludwig-Maximilian University, Munich, Germany

Thank you very much for an interesting work and novel approach to incentivise learners. I believe your article is well written and have no comments to make on the results.

 It might be interesting for the reader to learn what exactly you mean by "simulation centers do not have access to a formal ethical approval process" - is this the case for any simulation center, only for private ones, what are the rules governing this? While you clearly describe your adherence to all standards of good research and any ethics committee would approve your work, it might be worth adding a sentence on the exact reasons for not having institutional access to ethics.

Have any limitations of the research been acknowledged?

Yes

Is the work clearly and accurately presented and does it cite the current literature?

Yes

If applicable, is the statistical analysis and its interpretation appropriate?

Yes

Are all the source data underlying the results available to ensure full reproducibility?

Yes

Is the study design appropriate and is the work technically sound?

Yes

Are the conclusions drawn adequately supported by the results?

Yes

Are sufficient details of methods and analysis provided to allow replication by others?

Yes

Reviewer Expertise:

medical education, simulation based education, AR/VR training

I confirm that I have read this submission and believe that I have an appropriate level of expertise to confirm that it is of an acceptable scientific standard.

# Peer review: Reviewer response for version 1

**DOI:** 10.21956/mep.20506.r32054

**Published:** 2022-06-06

**Authors:** Fabrizio Consorti

**Affiliations:** 1 Department of Surgical Sciences, University of Rome La Sapienza, Roma, Italy

The manuscript reports on a quasi-experimental study on the use of a gamification app in learning of obstetrics anesthesia for residents.

 The study is based on a sound theoretical basis and is well designed. The results are clear and the conclusions are coherent.

 My only suggestions are:

- make explicit the learning outcomes. Since the assessment is based on MCQs, which is the taxonomic level of the outcomes? Knowledge of facts? Interpretation of facts? Ability to use knowledge to solve a problem?
- make clearer the mechanism of the league. You wrote that "Each participant represents a team, and the game is played with the opposing team ". Since the study group is composed of 137 participants, how can a league with 137 teams rely on 13 games?
- I found problematic the first sentence of the Discussion "The current young doctors’ generation utilizes games and simulations to create collaborative learning environments." Actually, FantaTraining does not create a "collaborative learning environment", because the team is composed of one member and the only interaction is with opponents: For an environment to be "collaborative", I'd expect that the team is composed of more members in interaction.

Have any limitations of the research been acknowledged?

Yes

Is the work clearly and accurately presented and does it cite the current literature?

Yes

If applicable, is the statistical analysis and its interpretation appropriate?

Yes

Are all the source data underlying the results available to ensure full reproducibility?

Yes

Is the study design appropriate and is the work technically sound?

Yes

Are the conclusions drawn adequately supported by the results?

Yes

Are sufficient details of methods and analysis provided to allow replication by others?

Partly

Reviewer Expertise:

Technology enhanced medical education

I confirm that I have read this submission and believe that I have an appropriate level of expertise to confirm that it is of an acceptable scientific standard.
